# GAIN: a multicenter pilot protocol for early initiation of enteral feeds in neonates with simple gastroschisis

**DOI:** 10.1186/s40814-026-01860-4

**Published:** 2026-06-15

**Authors:** Nicole T. Cacho, Anjali J. Cera, Daniel J. Tancredi, Minna M. Wieck, Jamie E. Anderson, Alexandra J. Leegwater, Cherry C. Uy, Yigit S. Guner, Daniel A. DeUgarte, Jennifer L. Rosenthal, Kara L. Calkins, Geoanna M. Bautista, University of California Fetal Consortium (UCfC), Twila Ong, Christa Bedford-Mu, Katy Wright, Kenzie Usher

**Affiliations:** 1 Department of Pediatrics, University of California Davis, Sacramento, CA 95817 USA; 2 Department of Pediatrics, Kaiser Permanente, Roseville, CA 95661 USA; 3 Department of Surgery, University of California Davis, Sacramento, CA 95817 USA; 4 Department of Pediatrics, University of California, Irvine, Irvine, CA 92697 USA; 5 Department of Surgery, Children’s Hospital of Orange County, Orange, CA 92868 USA; 6 Department of Surgery, University of California, Los Angeles, CA 90095 USA; 7 Department of Pediatrics, University of California, Los Angeles, CA 90095 USA

**Keywords:** Gastroschisis, Neonatal intensive care unit, Rare disease, Early feeds, Feasibility trial

## Abstract

**Background:**

Gastroschisis is a congenital abdominal wall defect characterized by severe gut dysmotility. These neonates often face lengthy hospital stays, prolonged parenteral nutrition dependence, and complications such as growth faltering, sepsis, and intestinal failure. Feeding practices for neonates with gastroschisis vary and emphasize extended bowel rest and delayed feeding initiation. Usual practices in determining timing to start feeds include awaiting return of bowel function which typically consists of low Replogle output volume, color changing from bilious to clear and/or passing stool. However, emerging studies suggest that early initiation of feeds through standardized protocols may improve outcomes. The Gastroschisis And Early Infant Nutrition (GAIN) Study aims to test the feasibility of an early feeding approach in neonates with simple gastroschisis.

**Methods:**

This two-site single-arm feasibility trial aims to enroll neonates with presumed simple gastroschisis over 30 months. The intervention has two main components: Replogle suction tube removal within 24 h of abdominal closure and starting small-volume human milk feeds within 48 h of abdominal closure. Feeding volume will be advanced as tolerated, following the University of California Fetal Consortium pathway, which is standard care. Feasibility outcomes include enrollment rate, protocol adherence, fidelity, and withdrawal rate. Exploratory outcomes include hospitalization length, time to full feeds, duration of parenteral nutrition, use of human milk, breastfeeding rates, and anthropometric measurements. Safety measures involve monitoring for feeding-related complications, infections, and unexpected surgical interventions.

**Discussion:**

This feasibility trial of early feeding initiation in neonates with gastroschisis is a critical step towards addressing the need for a standardized feeding approach. Findings will inform future clinical trials and challenge the need for prolonged bowel rest. Standardizing early feeding practices could reduce practice variation, improve gut motility, and the overall health of neonates with gastroschisis.

**Systematic review registration:**

NCT06878950

## Introduction

### Gastroschisis: clinical significance

Gastroschisis is a complex and costly birth defect characterized by the protrusion of fetal intestines through a hole in the abdominal wall. Neonates with gastroschisis carry significant morbidity, including prolonged hospitalization and parenteral nutrition (PN), high rates of growth faltering (30–55%), sepsis (19%), cholestasis (14–26%), and short bowel syndrome (7%) [1–4]. The mean cost for hospitalization for neonates with gastroschisis in the USA was $85,080 USD in 2019. Thirty-eight percent of these neonates were readmitted within the first year of age at an average cost of $186,873 USD [5]. Recent data suggest a decline in the prevalence of gastroschisis following decades of a steady increase. However, gastroschisis continues to affect 1.60 to 2.46 per 10,000 live births in the U.S. and primarily affects young mothers [6]. Despite this recent epidemiological shift, gastroschisis is the leading cause of pediatric intestinal failure, a multi-factorial disorder that carries substantial long-term consequences including malnutrition, stunted growth, central catheter-related infections and emboli, intestinal-failure-associated liver disease, neurodevelopmental impairment and psychosocial problems [1, 3, 7–9].

### Management challenges and variability in practice: need for standardization

Despite surgical repair, these neonates often have intestinal dysmotility, resulting in feeding intolerance, prolonged PN courses, and extended hospital stays. On average, neonates with gastroschisis receive their first feed at 15 days of life and require 25 PN days [1, 4, 10]. Management of neonates with gastroschisis varies widely across and within health systems. Clinicians lack evidence-based guidelines, particularly regarding feeding practices [11]. Usual practices in determining timing to start feeds include awaiting return of bowel function which typically consists of a combination of decreasing Replogle output volume, color changing from bilious to clear and/or passing stool. This variability in feeding may contribute to delays in achieving full enteral nutrition and prolong PN exposure and hospitalization.

**Fig. 1:**
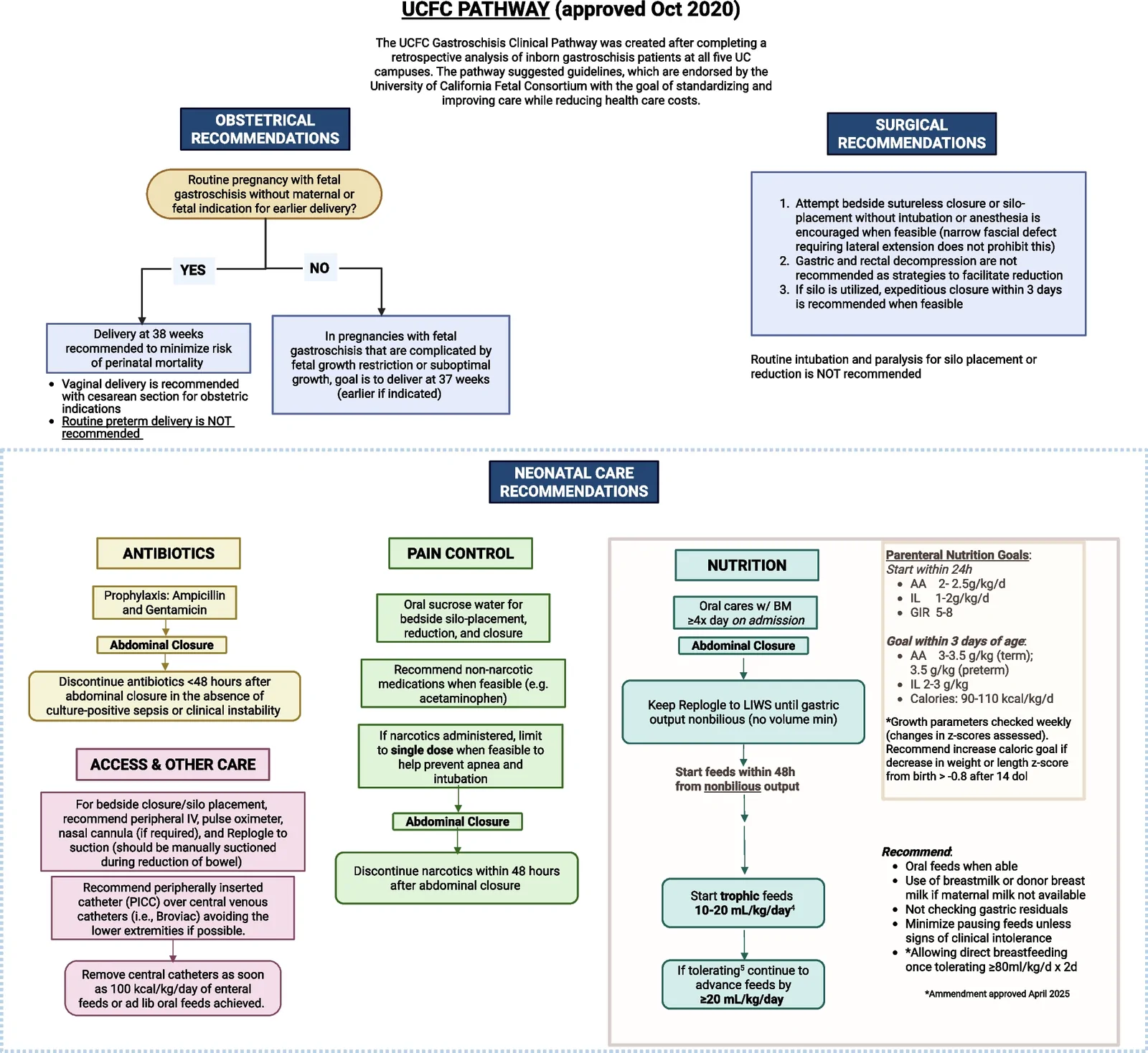
The UC Fetal Consortium (UCfC) Gastroschisis Clinical Pathway. Guidelines for managing neonates with gastroschisis currently used at sites across the UCfC. Created in BioRender. https://BioRender.com/mdt9g3m

The University of California Fetal Consortium (UCfC) implemented a standardized pathway (Fig. 1) across eight medical centers. Key advancements include consistent adherence to standardized protocols across institutions, resulting in statistically and clinically significant decreased ventilator days, decreased antibiotic days, and earlier initiation of enteral feeds [12]. While a standardized approach to nutrition improved growth, the duration of PN and length of stay (LOS) remained unchanged [4]. These results reflect unresolved barriers in transitioning to full enteral autonomy.

### Benefits of early enteral feeding practices

Growing evidence indicates that early introduction of enteral feeds in neonates with simple gastroschisis improves clinical outcomes [13–16, 18]. Traditionally, prolonged bowel rest was favored after surgical closure of the defect; however, mounting evidence suggests this practice may be unnecessary and potentially counterproductive to normal bowel functional maturation [13–16]. Meta-regression analyses in neonates with gastroschisis have demonstrated that each day delay in starting enteral feeds is associated with 1.4 additional days to reach full enteral feeds, 2.1 additional PN days, and 1.9 additional hospital days [17]. Early feeding, in neonates with and without gastroschisis, is also associated with improved weight gain and reduced intestinal inflammation, which may lead to a decrease in morbidity [13, 18, 19].

Despite accumulating evidence favoring early enteral feeding in gastroschisis, critical knowledge gaps hinder consensus guidelines [4, 11, 18, 20]. The absence of randomized controlled trials evaluating feeding protocols limits definitive recommendations, and institutional variability in existing protocols complicates comparative effectiveness analyses.

### Rationale for the Gastroschisis And Early Infant Nutrition Study (The GAIN Study)

The GAIN Study addresses a significant gap in the management of neonates with gastroschisis. Demonstrating the feasibility of a standardized approach to early feeding is a critical initial step towards helping to reduce unnecessary practice variation, shorten PN dependency and hospitalization, and improve both short and long-term outcomes for these vulnerable neonates and their families [18]. This protocol is based on a modified version of the gastroschisis feeding protocol implemented in Australia by Hobson et al*.* [18, 21]. This protocol was adapted in order to incorporate differences in current practices within the institutes of the UCfC. Ultimately, this study could have a significant impact on feeding practices for neonates with gastroschisis across the US and possibly worldwide.

## Objectives

We will test the feasibility of conducting a trial of initiating early introductory, gut-priming enteral feeds (1 mL every 4 h) within the first 48 h after abdominal closure in neonates with gastroschisis in a level III/IV neonatal intensive care unit (NICU). Feasibility will assess enrollment, protocol adherence, fidelity, withdrawal rate, and safety.

## Trial design

This study is a single-arm feasibility trial. All enrolled neonates will receive the intervention. This trial is not designed to test hypotheses regarding efficacy.

## Methods: participants, interventions and outcomes

### Study setting

This multi-site study will take place in two academic hospitals: University of California Davis and University of California Los Angeles. University of California Davis is a 121-bed children’s hospital within a large academic medical center in Northern California; the NICU has 49 beds and accepts patients from over 33 counties from a 65,000-mile region that includes Northern California, Southern Oregon, and Nevada. University of California, Los Angeles is a 90-bed children's hospital with a large academic medical center in Southern California; the NICU has 22 beds and accepts patients from over 18 cities or communities within and beyond Los Angeles County.

### Eligibility criteria

Eligible neonates will have presumed simple gastroschisis born at ≥ 34 weeks of gestation who are hemodynamically stable (not on pressors for blood-pressure support), born to mothers ≥ 16 years old and speak English or Spanish. Simple gastroschisis is defined as gastroschisis without associated intestinal complications—specifically, in the absence of atresia, necrosis, perforation, or volvulus. Deciding whether a neonate has presumed simple gastroschisis is a decision the GAIN study team, comprising of neonatologists and pediatric surgeons, will make in consultation with the primary medical and surgical teams. The pediatric surgeons will inspect the bowel, and the GAIN study team will gather this information and discuss any findings with the primary teams and make a preliminary determination of whether the infant qualifies. Exclusion criteria include complex gastroschisis at time of closure or solidified by definitive operative findings (any ischemic bowel, intestinal perforation, or obvious atresia with mesenteric defect), major congenital anomalies, ionotropic medications, neonates who are wards of the state, futile or redirection of care, participation in another interventional trial, and anyone deemed unfit for participation by study investigators or the primary medical team. A major congenital anomaly is a structural or functional defect present at birth that will have significant impact on the individual, including surgical, medical or serious cosmetic significance [22].

### Who will take informed consent

Trained study personnel will obtain informed consent antenatally when feasible or postnatally, prior to intervention initiation. Participation is voluntary and will not affect clinical care. The feeding protocol is implemented by the clinical team for enrolled participants; parents are not responsible for administering the intervention.

### Interventions

#### Explanation for the choice of comparators

This study is a single-arm feasibility trial, whereby every enrolled neonate is assigned to the intervention. Thus, a comparator group is not included. Because gastroschisis is rare, this single-arm design will optimize our ability to assess enrollment, protocol adherence, fidelity, withdrawal, and safety.

#### Intervention description

**Fig. 2:**
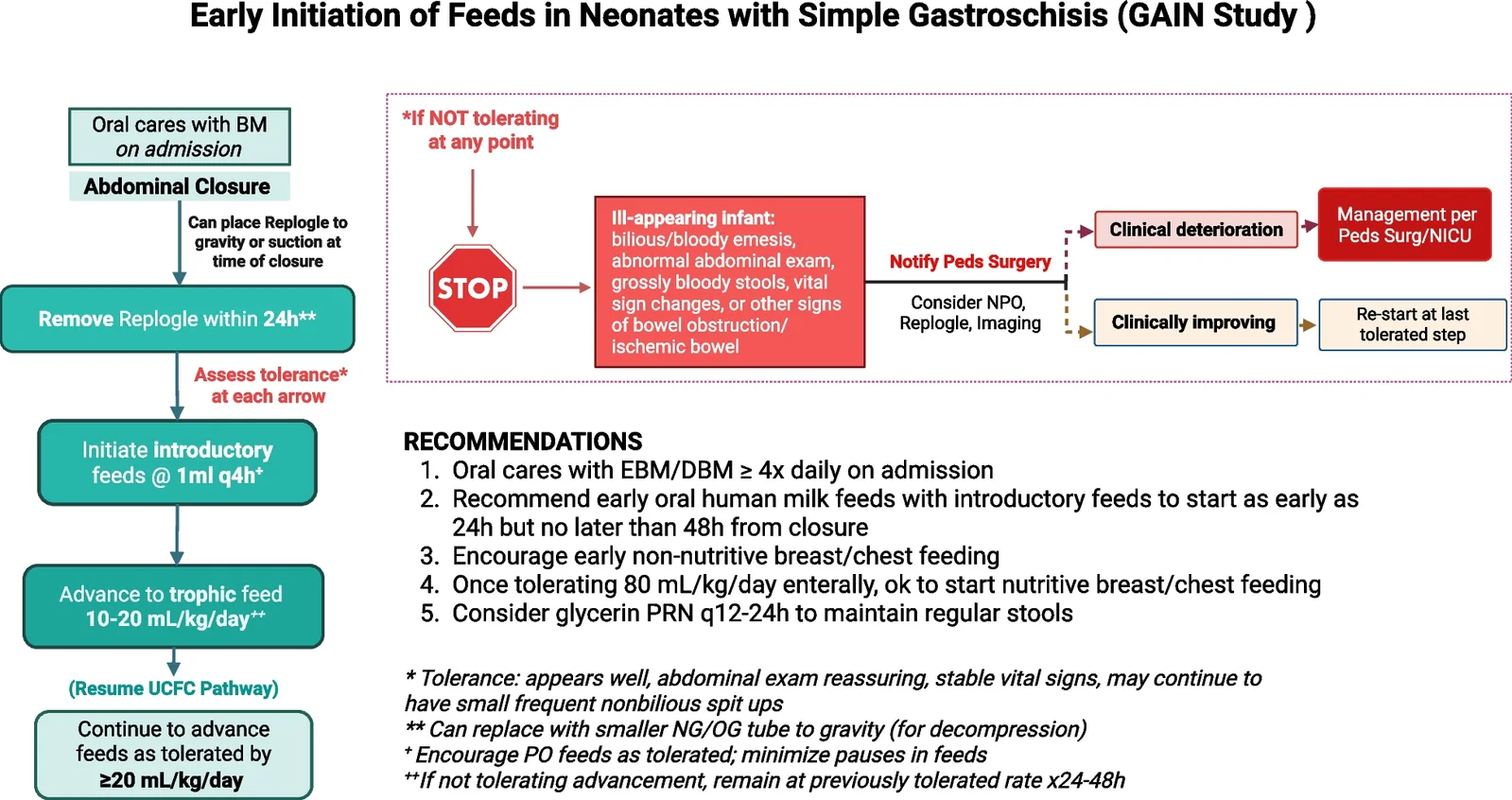
GAIN Study Feeding Protocol. Building on the UCfC Gastroschisis Nutrition Pathway, the primary change involves removing the Replogle within 24 h of abdominal closure and initiating introductory feeds to simulate peristalsis and continued maturation of the intestine. Created in BioRender. https://BioRender.com/eavktjs

The intervention includes implementing a Gastroschisis Early Feeding Protocol (Fig. 2). Specifically, for study patients, clinical practice is changed by removal of the Replogle suction tube within 24 h of abdominal closure and the initiation of introductory, gut-priming feeds (1 ml every 4 h) within 48 h of abdominal closure. The primary medical team can advance to trophic feeds (10–20 ml/kg/day) based on clinical tolerance, and then resume the standard care pathway, which advances feeds by approximately 20 ml/kg/day as tolerated. If the parents decline to consent, neonates will follow our current standard UCfC Gastroschisis Pathway (Fig. 1). Direct breastfeeding is allowed for both study patients and non-study patients if the neonate is tolerating enteral feeding of 80 ml/kg/day for at least 2 days. Our study defines feeding tolerance as an infant who is well appearing, and has a reassuring abdominal exam, nonbloody stools, and stable vital signs. Infants may have small frequent nonbilious, nonbloody spit ups. If the infant is not tolerating feeds, Pediatric Surgery should be notified and feedings should be suspended. A Replogle may be placed and/or imaging may be required (Fig. 2).

#### Criteria for discontinuing or modifying allocated interventions

The intervention may be discontinued or modified for the following reasons: parent request, classification as complex gastroschisis (finding of intestinal perforation, atresia, necrosis), feeding intolerance, ill appearing, need for mechanical ventilation, vasoactive medications, or clinician judgment.

#### Strategies to improve adherence to interventions

Strategies to improve adherence include educational sessions for clinical staff, feedback from medical teams, appointment of champions, pre-delivery huddles, visual paper reminders at bedside and weekly check-ins. Adherence will be monitored using protocol checkpoints highlighted in study materials, such as confirming Replogle tube removal within 24 h and initiating enteral feeds within 48 h of abdominal closure.

#### Relevant concomitant care permitted or prohibited during the trial

No concomitant medications or interventions are prohibited. All concomitant care will be documented throughout the study.

#### Provisions for post-trial care

Provisions for ancillary and post-trial care are not planned as part of this study.

### Outcomes

#### Feasibility objective outcomes

We will test the feasibility of implementing an early feeding protocol for neonates with gastroschisis. Feasibility will be assessed through:

1. Objective 1 Enrollment (primary feasibility objective): obtain consent from ≥ 80% of eligible neonates’ parents/guardians from the prenatal period to within the first 24 h of abdominal closure.
2. Objective 2 Protocol adherence: ≥ 85% of enrolled neonates receive introductory feeds within 48 h of abdominal closure.
3. Objective 3 Fidelity: feeding protocol adherence with ≥ 90% of predefined protocol steps.
4. Objective 4 Withdrawal: < 10% of enrolled neonates withdraw.

#### Exploratory outcomes

Data will be collected prospectively via electronic health records. These measures will be compared to a historical cohort using the existing UCFC database, which has been previously approved by IRB protocol # 2216195–2 at UC Davis, and IRB protocol # 24–6179 at UCLA.

Exploratory clinical endpoints will include (1) length of hospitalization (days), (2) time to initiation of feeds from final abdominal closure (days), (3) time to full feeds (100 mL/kg/day or PO ad libitum whichever comes first) from final abdominal closure (hours), (4) duration of parenteral nutrition (days), (5) days of any human milk which includes parent’s own milk and/or donor breast milk during hospitalization, (6) any direct breastfeeding during hospitalization, (7) any direct breastfeeding at discharge (defined as 48 h prior to hospital discharge) (8) any use of parent’s own milk at discharge (defined as 48 h prior to hospital discharge), (9) growth parameters (delta-z-scores for weight and length (discharge–admission measurements).

The length of hospitalization refers to the total number of days spent in the hospital during the initial birth hospitalization. Generally, this will be days in the neonatal intensive care unit (NICU) but can also be days in the pediatric intensive care unit (PICU) or pediatric wards. This will be measured via midnight census data from electronic health records (EHR). Feeding progression covers the initiation of feeds, defined as time to first feed and time to full feeds, both in days. The duration of PN is measured as the number of days of PN. Rates of direct breastfeeding are defined as direct breastfeeding 48 h before discharge. Outcomes of adverse events include rates of necrotizing enterocolitis (> Stage II), sepsis, abdominal compartment syndrome, additional abdominal surgeries (excluding primary closure), and mortality. Medical errors and adverse events will be obtained via solicited reports and review of EHR data.

### Participant timeline

We will conduct the feasibility study over a 36-month period. Participation in the study and interventions will occur in the first 30 months.

### Sample size

We plan to enroll 20 neonates with gastroschisis from the University of California Davis (UCD) and the University of California Los Angeles (UCLA). This sample size was determined based on availability and feasibility. We estimate that approximately 10 neonates per year will meet the eligibility criteria, yielding a denominator of approximately 40 for the consent outcome (feasibility objective 1) and that the number of enrolled patients will be at least 20 for the proportions used for feasibility objectives 2 to 4. These two denominators will allow us to estimate these proportions with error margins less than 16 and 22 percentage points, respectively.

### Recruitment

To achieve adequate participant enrollment, strategies include active screening of eligible patients at each site, clear communication about the study, and collaboration with healthcare providers to identify potential participants. The study team will streamline the consent process, provide educational materials, and hold regular meetings to address recruitment challenges. Recruitment progress will be monitored, and additional outreach efforts will be made as necessary to reach the target sample size.

### Data collection and management

#### Plans for assessment and collection of outcomes

Feasibility objectives will be assessed by tracking the number of neonates approached, enrollment rates, milestone achievement (such as initiation of feeds within 48 h of abdominal closure), and withdrawal rates. Data for the intervention group will be collected prospectively from the electronic health record (EHR) prenatally and through the initial hospital discharge.

#### Plans to promote participant retention and complete follow-up

To promote participant retention and ensure thorough follow-up, the study team will regularly communicate with participants’ families and healthcare providers, and deliver clear details about study procedures. For participants who discontinue or stray from the intervention protocol, essential outcome data—like clinical status, growth metrics, and pertinent hospitalization results—will still be gathered whenever possible. These strategies aim to optimize data completeness and reduce loss to follow-up.

#### Data management

Data for the study will be collected from the EHR and input directly into and stored in REDCap and handled by the research staff at the site. All data will be de-identified.

#### Confidentiality

Personal information about potential and enrolled participants will be collected using secure, study-specific data collection forms and entered into password-protected electronic databases. All study-related information will be stored securely at each site, with physical documents kept in locked files and electronic data protected by access controls. Identifiable information will be accessible only to authorized study personnel and will not be included in any reports, publications, or shared datasets. Data shared outside the study team will be de-identified to protect participant confidentiality. These measures will be maintained before, during, and after the trial to ensure ongoing protection of participant privacy in accordance with regulatory and ethical standards.

### Statistical methods

#### Statistical methods for primary and secondary outcomes

For each feasibility outcome, we will report proportions with 95% confidence intervals. For rate-based outcomes where the denominators can vary by patient (e.g., daily breastfeeding receipt), we will report the overall rate (e.g., the denominator-weighted mean rate). For other binary and continuous outcomes, we will report sample proportions and means, respectively. All estimates will be reported with appropriate 95% confidence intervals.

We will review the data after one subject completes the study, again after five subjects have completed the study, and finally after 10 subjects have completed the study at each of the two sites. We anticipate completion of primary analysis within 3 years.

#### Interim analysis

One interim analysis is planned at the midpoint of participant enrollment to assess safety and feasibility outcomes. The Data Monitoring Committee (DMC) will have exclusive access to interim results and will review recruitment and study data to determine if there are any safety concerns and/or other concerns. The DMC will make recommendations to the study investigators regarding continuation, modification, or termination of the trial based on these findings. Final decisions will be made by the study investigators in consultation with the DMC.

#### Methods for additional analyses, including comparisons to historical controls

We recognize that accounting for specific confounders, such as site, gestational age, birth weight, sex, and race/ethnicity, is important. If feasible, we may consider performing sub-analyses or accounting for these variables in specific models. However, we recognize that our sample size is small and our results most likely will not be generalizable.

For comparisons to historical controls on the study variables described earlier, we anticipate being able to obtain patient-level data from the historical controls, permitting us to use linear and logistic regression to estimate adjusted mean differences or adjusted odds ratios, respectively, with 95% confidence intervals for the current vs. historical control comparisons, adjusting for study site. Continuous outcomes may be log-transformed to improve the validity of the normality assumption, with the Van Elteren test (stratified Wilcoxon Mann–Whitney) used as a backup if parametric tests are not appropriate. If patient-level data from the historical controls is not available but means and/or proportions are available, we will instead estimate stratified means and/or proportions for the current cohort with 95% confidence intervals, permitting comparisons to the historical means and proportions.

#### Methods in analysis to handle protocol non-adherence and any statistical methods to handle missing data

The primary analysis population will include all enrolled participants, regardless of protocol adherence, following the intention-to-treat principle. For participants who discontinue or deviate from the protocol, available outcome data will still be collected and included in the analysis to minimize attrition bias. For each specific feasibility outcome, it will be counted as a success if it was known to happen and a failure if either it was known to not happen or its status is unknown.

#### Adverse event reporting and harms

Potential adverse events will be discussed and categorized (based on severity, expectedness and relatedness) by the principal investigators.

## Discussion

This prospective study of a standardized feeding protocol for neonates with gastroschisis faces several operational challenges. Recruitment may be limited by the rarity of the condition and the need to approach families during a stressful time. To address this, we have streamlined screening and consent processes and provided targeted staff education.

Coordinating data collection across two centers requires clear communication and standardized procedures. Regular joint meetings and routine data audits are in place to ensure data quality. Protocol adherence is promoted through staff training, site champions, and visual reminders.

Given the vulnerable neonatal population, all protocol changes receive IRB approval and participant safety is closely monitored. While the sample size and single-region setting may limit generalizability, the study will provide valuable insights into feeding management for neonates with gastroschisis and inform future research.

## Trial status

IRB Protocol Version Date is January 31, 2025. Recruitment started February 20, 2025. The anticipated date of recruitment completion is February 1, 2028. Registered March 10, 2025 retrospectively registered.

## Data Availability

After publication, de-identified participant data will be available to qualified researchers upon request, subject to a data-use agreement, study team review, and IRB approval. Access will be granted within 12 months for requests meeting scientific and ethical standards.

## Abbreviations

NICU: Neonatal intensive care unit
UCD: University of California Davis
UCLA: University of California Los Angeles
UCfC: University of California Fetal Consortium
PN: Parenteral Nutrition
DMC: Data Monitoring Committee
EMR: Electronic Medical Record
GCP: General Clinical Practice
